# Parathyroid hormone‐like hormone plays a dual role in neuroblastoma depending on PTH1R expression

**DOI:** 10.1002/1878-0261.12542

**Published:** 2019-07-19

**Authors:** Marta García, Carlos Javier Rodríguez‐Hernández, Silvia Mateo‐Lozano, Sara Pérez‐Jaume, Eliana Gonçalves‐Alves, Cinzia Lavarino, Jaume Mora, Carmen de Torres

**Affiliations:** ^1^ Developmental Tumor Biology Laboratory Institut de Recerca Sant Joan de Déu Esplugues de Llobregat Spain; ^2^ Department of Haematology and Oncology Hospital Sant Joan de Déu Barcelona Esplugues de Llobregat Spain

**Keywords:** epidermal growth factor receptor, invasion, neuroblastoma, parathyroid hormone type 1 receptor, parathyroid hormone‐like hormone

## Abstract

We have previously reported the expression of parathyroid hormone‐like hormone (PTHLH) in well‐differentiated, Schwannian stroma‐rich neuroblastic tumors. The aim of this study was to functionally assess the role of PTHLH and its receptor, PTH1R, in neuroblastoma. Stable knockdown of *PTHLH* and *PTH1R* was conducted in neuroblastoma cell lines to investigate the succeeding phenotype induced both *in vitro* and *in vivo*. Downregulation of *PTHLH* reduced MYCN expression and subsequently induced cell cycle arrest, senescence, and migration and invasion impairment in a *MYCN*‐amplified, *TP53*‐mutated neuroblastoma cell line. These phenotypes were associated with reduced tumorigenicity in a murine model. We also show that PTHLH expression is not under the control of the calcium‐sensing receptor in neuroblastoma. Conversely, its production is stimulated by epidermal growth factor receptor (EGFR). Accordingly, irreversible EGFR inhibition with canertinib abolished PTHLH expression. The oncogenic role of PTHLH appeared to be a consequence of its intracrine function, as downregulation of its receptor, PTH1R, increased anchorage‐independent growth and induced a more undifferentiated, invasive phenotype. Respectively, high *PTH1R* mRNA expression was found in *MYCN* nonamplified primary tumors and also significantly associated with other prognostic factors of good outcome. This study provides the first evidence of the dual role of PTHLH in the behavior of neuroblastomas. Moreover, the identification of EGFR as a transcriptional regulator of PTHLH in neuroblastoma provides a novel therapeutic opportunity to promote a less aggressive tumor phenotype through irreversible inhibition of EGFR tyrosine kinase activity.

**Abbreviations**
FBS: fetal bovine serum
GEO: gene expression omnibus
IC_50_: half maximal inhibitory concentration
INSS: international neuroblastoma staging system
PCR: polymerase chain reaction
SDS/PAGE: sodium dodecyl sulfate polyacrylamide gel electrophoresis
shNT: nontargeting shRNA
shRNA: short hairpin RNA
siNT: nontargeting siRNA
siRNA: small interfering RNA

## 1 Introduction

Neuroblastic tumors comprise a heterogeneous group of malignancies derived from precursor cells of the peripheral nervous system (Cheung and Dyer, 2013). Poor prognosis is associated with age at diagnosis older than 18 months, metastases or undifferentiated histopathology, and amplification of the oncogene *MYCN*. Although disseminated neuroblastomas in infants undergo spontaneous regression, a high proportion of older patients with metastatic neuroblastomas succumb to disease despite the multimodal treatment currently considered standard of care (Maris, 2010). However, molecular events underlying neuroblastoma metastases are still poorly understood, thus precluding the identification of novel therapeutic strategies for these patients.

*MYCN* amplification was the first genetic prognostic factor identified in these developmental malignancies (Brodeur *et al*., 1984). Besides its role during development through direct or indirect regulation of numerous genes, exerts crucial functions in virtually every mechanism responsible for the aggressive behavior of neuroblastomas (He *et al*., 2013; Huang and Weiss, 2013). Accordingly, *MYCN* silencing has been reported to induce cell cycle arrest, apoptosis and cytodifferentiation of neuroblastoma cells (Janardhanan *et al*., 2009), and pharmacological inhibition of MYCN activities is being actively pursued (Gustafson and Weiss, 2010; Henssen *et al*., 2016).

Over the past few years, we have reported that the calcium‐sensing receptor (CaSR) acts as a tumor suppressor in neuroblastoma (Casalà *et al*., 2013; Rodríguez‐Hernández *et al*., 2016). CaSR is a G protein‐coupled receptor critically required to regulate parathyroid hormone (PTH) secretion and calcium homeostasis (Brennan *et al*., 2013). In some physiological (VanHouten *et al*., 2004) and neoplastic (Sanders *et al*., 2000) contexts, CaSR activation results in increased production of parathyroid hormone‐like hormone (PTHLH), a peptide responsible for malignant hypercalcemia (Suva *et al*., 1987) that shares structural homology with the N‐terminal region of PTH. PTHLH has been reported to act as an autocrine, paracrine and intracrine factor (McCauley and Martin, 2012). In cancer, *in vitro* and *in vivo* evidences indicate that PTHLH promotes tumor initiation, growth and metastatic spread (Li *et al*., 2011; Park *et al*., 2013). Interestingly, its role as a mitogenic and survival factor in two different contexts has been associated with upregulation of the transcriptional factor MYC (Bhatia *et al*., 2009; Hochane *et al*., 2013).

Our initial data showed that PTHLH expression was detected in all groups of neuroblastic tumors, but the highest levels of expression were found in ganglioneuroblastomas and ganglioneuromas, two benign histologic subgroups that contain a high proportion of glial, Schwannian‐like cells (de Torres *et al*., 2009). Of note, PTHLH and its receptor, PTH1R, are co‐expressed in the rat peripheral nervous system, where, upon sciatic nerve injury, upregulation of PTHLH induces a notable increase in the number of Schwann cells (Macica *et al*., 2006). Furthermore, intracrine PTHLH has been shown to induce proliferation, while its paracrine action through PTH1R, promotes the opposite phenotype in vascular smooth muscle cells (Massfelder *et al*., 1997).

On the other hand, epidermal growth factor (EGF), a well‐established regulator of PTHLH production in several epithelial cancers (Cramer *et al*., 1996), supports the proliferation of neural precursor cells but also induces their differentiation toward the oligodendrocyte lineage (Gonzalez‐Perez *et al*., 2009). Furthermore, EGFR family of receptors is expressed in neuroblastoma cell lines and tumors (Ho *et al*., 2005) where they promote tumor growth (Ho *et al*., 2005; Richards *et al*., 2010) and MYCN induction (Hossain *et al*., 2012).

All these data considered, the aim of this work was to functionally analyze the role of PTHLH and its receptor, PTH1R, in neuroblastoma. Given that our results indicated that their expression is necessary for neuroblastoma cell invasion and that PTHLH knockdown reduces MYCN expression, we sought to identify the mechanism responsible for PTHLH production in neuroblastoma. As expected, EGFR was found to control PTHLH expression, thus making it clinically feasible to target this molecule specifically in neuroblastic primary tumors.

## 2 Materials and methods

### 2.1 Patients and primary tumor samples

Forty‐four snap‐frozen neuroblastic tumors obtained at diagnosis at Hospital Sant Joan de Déu (Barcelona, Spain) were analyzed. Selection criteria included histopathologic diagnosis and availability of frozen tumor fragments of good quality (viable tumor cell content > 70%) and quantity. Informed written consent was obtained from patients/parents/legal guardians and procedures were approved by the Institutional Review Boards. The study methodology was conformed to the standards set by the Declaration of Helsinki. Tumors were classified as: undifferentiated neuroblastoma, differentiating or poorly differentiated neuroblastoma and ganglioneuroblastoma/ganglioneuroma. Also, age at diagnosis, clinical stage (International Neuroblastoma Staging System, INSS), clinical risk, *MYCN* amplification status, and time to follow‐up were recorded. Patients were classified as high risk if they were stage 4 or *MYCN*‐amplified, and low risk otherwise. Tumors were designated as *MYCN*‐amplified if they had ≥ 10 *MYCN* copies. Four datasets were included in the study to validate results obtained in our cohort. These datasets are available at the NCBI Gene Expression Omnibus (GEO) data repository (Table S1).

### 2.2 Cell lines

Eight neuroblastoma cell lines (IMR5, LA‐N‐1, LA1‐55n, LA1‐5s, SH‐SY5Y, SK‐N‐AS, SK‐N‐JD, and SK‐N‐LP), osteosarcoma cell line U2OS and HEK293T cell line were obtained from the repository at Hospital Sant Joan de Déu (Barcelona, Spain). Unless otherwise specified, cells were grown as described elsewhere (Rodríguez‐Hernández *et al*., 2016). Mycoplasma PCR tests were monthly performed. Characterization of neuroblastoma cell lines included analysis of *MYCN* status (de Torres *et al*., 2009), *TP53* sequence and authentication by STR profiles.

### 2.3 Reagents

Animal‐Free Recombinant Human EGF was obtained from PreProtech (London, UK). Stock solutions were prepared in water and stored at −80 °C. Canertinib (CI‐1033, Selleckchem, Houston, TX, USA), U0126 (Selleckchem) and LY294002 (Calbiochem, Darmstadt, Germany) were prepared in DMSO and stored at −20 °C. pTH‐related protein (1–34) amide trifluoroacetate salt [PTHLH (1–34), Bachem, Switzerland] was prepared in 0.1% BSA, 1 mm HCl at 250 μm and stored at −80 °C.

### 2.4 RNA isolation, cDNA synthesis, PCR and qPCR

Total RNA was isolated using TriReagent (Sigma‐Aldrich, St Louis, MO, USA). Retrotranscription, PCR and qPCR were carried out as described (Casalà *et al*., 2013; de Torres *et al*., 2009). Real‐time PCR runs were performed in a QuantStudio 6 (Applied Biosystems, Forster City, CA, USA) using gene‐specific Assays on Demand and Taqman Universal PCR Master Mix or specific primers and SYBRGreen (Applied Biosystems) (Table S2).

### 2.5 Immunoblots

Cells were lysed in 10 mm Tris/HCl pH 6.8, 1 mm EDTA, 150 mm NaCl, 1% SDS. Thirty to eighty μg proteins were electrophoresed in 8–15% SDS/PAGE and transferred onto nitrocellulose membranes. To quantify secreted MMP‐2 protein, cells were seeded in serum‐free medium for 24 h. Conditioned media were collected, centrifuged at 250 ***g*** at 4 °C for 5 min. Protein content of the supernatants was electrophoresed in 8% SDS/PAGE and transferred onto nitrocellulose membranes. Incubation with primary antibodies (Table S3) was followed by secondary antibody IRDye680RD goat anti‐mouse IgG (Li‐COR #926‐68070) or IRDye800CW goat anti‐rabbit IgG (Li‐COR #926‐32211). Blots were visualized and quantified using Li‐COR Odyssey system (Li‐COR Biosciences, Lincoln, NE, USA).

### 2.6 shRNA knockdown of PTHLH and PTH1R

Human *PTHLH* and *PTH1R* genes were stably silenced using shRNA‐based MISSION technology (Sigma‐Aldrich) (Table S2) with lentiviral particles. HEK293T were transfected with the packaging plasmids (RRE, Rev and VSV‐G), and the correspondent shRNA vector and lentiviral particles were collected from the medium for 2 days. Neuroblastoma and osteosarcoma cells were infected with the produced lentiviruses and selected with puromycin (Sigma‐Aldrich). As a control, a MISSION Non‐Target shRNA control vector (Sigma‐Aldrich) was used.

### 2.7 siRNA mediated knockdown of PTHLH

Human *PTHLH* gene was transiently silenced in LA‐N‐1 neuroblastoma cells using Dharmacon ON‐Targetplus siRNA technology (GE Healthcare Life Sciences, Logan, UT, USA). Cells were seeded at a density of 3 × 10^5^ in 6‐well plates. Next day, they were transfected with 2 μL/well of DharmaFECT (GE Healthcare Life Sciences) and a 25 nM mixture of 4 siRNA against human *PTHLH* or a nontargeting (NT) pool following manufacturer's indications.

### 2.8 Cell viability assays

Cells were plated into 96‐well plates. Six replicate wells were seeded for each cell line and condition. Cell viability and IC_50_ were measured with CellTiter^96^ Aqueous Cell Proliferation Assay (Promega, Madison, WI) according to the manufacturer's indications at indicated times. The IC_50_ was calculated at 72 h with GraphPad Prism software (San Diego, CA, USA).

### 2.9 Cell cycle assays

Cells were seeded in RPMI‐1640 10% FBS. Next day, they were synchronized through serum deprivation for 16 h and then allowed to re‐enter cell cycle in complete medium. Cells were trypsinized, collected, fixed with cold 70% ethanol and stained with propidium iodide (Sigma‐Aldrich) at 0 and 24 h for cell cycle analysis. Cells were analyzed by flow cytometry on a NovoCyte System and results were processed with novoexpress software (ACEA Biosciences, San Diego, CA, USA).

### 2.10 Senescence‐associated β‐galactosidase assay

Cells were seeded in 6‐well plates. Forty‐eight hours later, β‐galactosidase assay was performed using a histochemical staining kit (Sigma‐Aldrich) according to manufacturer's instructions.

### 2.11 *In vitro* assays and PTHLH production

Cells (10^6^) were plated in p60 cell plates. Twenty‐four hours later, media were replaced with RPMI‐1640 10% dialyzed FBS for 24 h. Following indicated treatments, floating and adherent cells were collected and processed for RNA isolation or immunoblotting.

To examine the effect of *CaSR* activation on *PTHLH* production in CaSR‐expressing neuroblastoma cells, we proceeded as described elsewhere (Rodríguez‐Hernández *et al*., 2016).

### 2.12 Wound healing assay

Cells (2 × 10^6^) were seeded in 6‐well plates and allowed to reach 90% confluence. A wound was made by scratching the cell monolayer using a sterile pipette tip. Cultures were then rinsed with PBS and incubated in complete or serum‐free media at 37 °C in a 5% CO_2_ incubator. Wound area relative to time 0 was calculated at 24 and 48 h later using imagej ( National Institutes of Health, Bethesda, MD, USA).

### 2.13 Invasion assay

Cell invasion analyses were performed using 8 μm pore size, 24‐well Falcon Cell culture inserts (Corning, New York, NY, USA) covered with 70 μL of Matrigel (Corning) diluted 1 : 10 in serum‐free media. Cells were seeded in 200 μL of serum‐free media in the upper compartment, whereas the lower compartment was filled with complete medium containing 10% FBS as a chemoattractant. Forty‐eight hours later, invasive cells were fixed with 4% paraformaldehyde for 20 min and stained with 1% crystal violet. The number of stained cells was counted under the microscope.

### 2.14 Anchorage‐independent growth assay

LA‐N‐1 (5 × 10^4^) or IMR5 (2 × 10^5^) cells were seeded in RPMI‐1640 10% FBS containing 0.3% noble agar (BD Difco, Franklin Lakes, NJ, USA) and plated on top of 0.6% noble agar in the same medium. Two weeks later, growing colonies were stained with nitrotetrazolium blue chloride (1 mg**·**mL^−1^; Sigma‐Aldrich) and visible colonies were scored.

### 2.15 Gelatin zymography

Metalloproteinase activity was analyzed by gelatin zymography. Cells (2 × 10^6^) were seeded in serum‐free medium for 48 h. Conditioned media were centrifuged to clarify debris and electrophoresed on SDS 8% polyacrylamide gels containing 250 μg**·**mL^−1^ of gelatin (Sigma‐Aldrich). After electrophoresis, gels were incubated for 1 h in 2.5% Triton X‐100 and left overnight at 37 °C in Collagenase buffer (50 mm Tris/HCl pH 7.6; 5 mm CaCl_2_; 0.2 m NaCl). As a loading control, conditioned media were electrophoresed in parallel on SDS 8% polyacrylamide gels. Then, gels were stained with 0.1% Coomassie Brilliant Blue and destained with 10% acetic acid in 40% methanol. The gelatinolytic activity was identified as transparent band in the Coomassie Brilliant Blue‐staining background. Quantification of the intensity of the bands was done using imagej software (NIH).

### 2.16 Mouse xenograft models

Procedures were approved by the Institutional Animal Research Ethics Committee (CEEA nº 553/16). Sh‐derivatives from LA‐N‐1 (sh44, sh45, and shNT) and IMR5 (sh45, sh46, and shNT) (10^7^) were resuspended in PBS:Matrigel (Corning) and subcutaneously inoculated in both flanks of four to six‐week‐old female athymic Nude‐Foxn1 *nu*/*nu* mice (Charles River, Wilmington, MA, USA). Tumors were measured thrice a week using a digital caliper and allowed to grow until 1000 mm^3^. Tumor volume was calculated as L × W^2^/2 in which ‘L’ indicates length in mm and ‘W’ indicates width in mm. At the end of the experiment, tumors were excised and kept frozen in liquid nitrogen. The experiment was repeated twice with 5 mice (10 tumors) in each group.

### 2.17 Statistical analysis

Comparisons of quantitative variables among groups were performed by Student *t*, Mann–Whitney, or Kruskal–Wallis tests, depending on the number of groups and the distribution of the data. Statistical analyses were carried out using *r* software version 3.4.2 (Team, 2017) and graphpad prism. *P *<* *0.05 was considered statistically significant.

## 3 Results

### 3.1 *PTHLH* knockdown reduces neuroblastoma tumor growth *in vitro* and *in vivo*

Expression of *PTHLH* mRNA and protein was determined in neuroblastoma cell lines with different *MYCN* and *TP53* status. As shown in Fig. S1a, PTHLH was expressed in all neuroblastoma cell lines. Overall differences between PTHLH mRNA and protein expression levels can be explained due to the complexity of the human PTHLH gene regulation, which is transcribed by three functionally distinct promoters that lead to primary mRNA transcripts with different stabilities.

**Figure 1:**
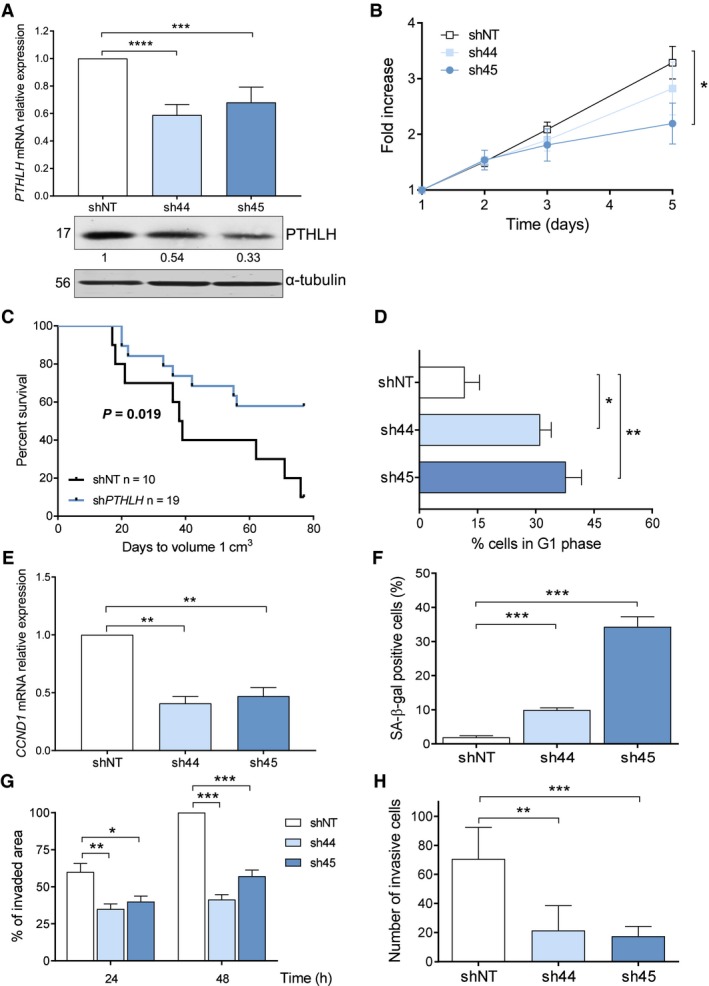
Knockdown of *PTHLH* reduces *in vitro* and *in vivo* tumor growth as well as invasion and migration capabilities in neuroblastoma cells. (A) *PTHLH* mRNA relative expression and protein levels in LA‐N‐1 cells stably transduced with shRNA against *PTHLH* or a control nontargeting (NT) shRNA analyzed by RT‐qPCR and western blot, respectively. Band intensities were quantified relative to α‐tubulin and normalized relative to shNT. (B) Cell viability of LA‐N‐1 sh‐*PTHLH*‐derivative cells measured with CellTiter^96^ Aqueous Cell Proliferation Assay. *N* = 6. (C) LA‐N‐1 sh‐derivatives (sh44, sh45 and shNT) (10^7^) subcutaneously inoculated in four‐ to six‐week‐old female athymic nude mice. The log‐rank statistic was used to compare the tumor time to reach 1 cm^3^ between groups. (D) Cell cycle analyses from LA‐N‐1 sh‐*PTHLH*‐derivative cells conducted at 8 h. *N* = 3. (E) *CCND1* mRNA relative expression levels analyzed by RT‐qPCR in LA‐N‐1‐synchronized cells. *N* = 5. (F) Senescence‐associated β‐galactosidase activity in LA‐N‐1 sh‐*PTHLH*‐derivative cells. Results are expressed as the average percentage of stained cells in ten independent counts. *N* = 3. (G) Wound healing assay conducted with LA‐N‐1 sh‐*PTHLH*‐derivative cells (2 × 10^6^). Wound area was calculated at 24 and 48 h later relative to time 0. *N* = 6. (H) Transwell invasion assay in LA‐N‐1 sh‐*PTHLH*‐derivative cells. Invasive cells were counted at 48 h. *N* = 3. Error bars represent SEM. *P <* *0.05, **P <* *0.01, ***P <* *0.001, Mann–Whitney *U*‐test (a, b, d–f) or two‐tailed Student's *t*‐test (g, h).

To evaluate the function of PTHLH in this developmental tumor, stable knockdown was conducted by short hairpin RNA (shRNA) in LA‐N‐1 and IMR5 cell lines (Figs 1A and S1b, respectively). Different shRNA‐generated clones against the same target were tested, and only congruent phenotypic tendencies were considered.

Most derivatives showed increased cell death upon stable silencing of *PTHLH* and among surviving sh‐*PTHLH* clones, those derived from LA‐N‐1 cell line, but not from IMR5, exhibited a slower *in vitro* growth rate (Figs 1B and S1c, respectively). In agreement with this result, *in vivo* tumor growth of those LA‐N‐1 sh‐*PTHLH* derivatives was significantly inhibited (Fig. 1C). In contrast, IMR5 sh‐*PTHLH* derivatives and controls showed a similar tumorigenicity (Fig. S1d).

As shown in Fig. 1D, reduced *in vitro* and *in vivo* growth of LA‐N‐1 sh‐*PTHLH* clones was a consequence, at least partially, of cell cycle arrest, as sh‐*PTHLH* clones were found to accumulate in G_0_/G_1_ phase for a longer time when compared to shNT cells following serum deprivation (Fig. S1e). Knockdown clones also displayed significantly lower cyclin D1 *(CCND1)* mRNA levels compared to shNT control (Fig. 1E). Interestingly, LA‐N‐1 sh‐*PTHLH* clones also exhibited a significant increase in senescence‐associated β‐galactosidase‐positive cells when compared to controls (Fig. 1F).

### 3.2 *PTHLH* knockdown significantly decreases neuroblastoma cell invasion and migration

In order to get further insights into the role of PTHLH in the tumorigenicity of neuroblastoma, we explored whether stable knockdown of *PTHLH* expression affects the invasive and migratory capacities of those cells. Wound healing assays, with serum‐free media to inhibit proliferation, were performed. However, cell death in sh‐*PTHLH* derivatives was too high to be evaluable. Wound healing assays in complete media showed that stable downregulation of *PTHLH* expression was associated with significantly slower migratory capacities both in LA‐N‐1 and IMR5 sh‐clones compared with controls (Figs 1G and S1f, respectively). This could be partly due to growth rate, but it was observed only in LA‐N‐1 cell line and not in IMR5 (Figs 1B and S1c, respectively). In transwell assays, only LA‐N‐1 sh‐*PTHLH* clones showed significantly reduced invasive capacities when compared to control cells (Figs 1H and S1 g).

### 3.3 Stable downregulation of *PTHLH* reduces *MYCN* expression and function

**Figure 2:**
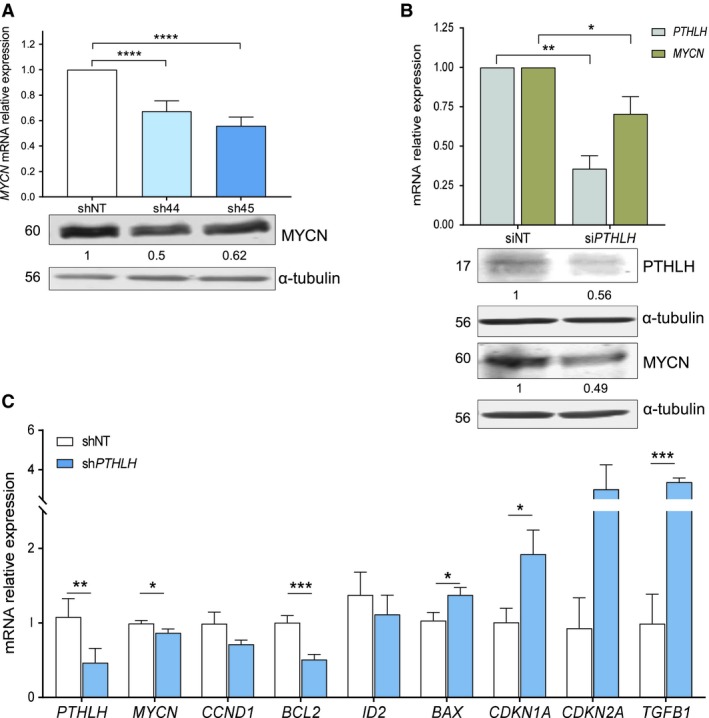
Knockdown of *PTHLH* reduces *MYCN* expression. (A) *MYCN* mRNA relative expression and protein levels in LA‐N‐1 sh‐*PTHLH*‐derivative cells. Band intensities were quantified relative to α‐tubulin and normalized relative to shNT. Data from RT‐qPCR are *N* = 10 and blots shown are representative of *N* = 3. (B) *PTHLH* and *MYCN* mRNA relative expression levels 24 h after transient downregulation of *PTHLH* expression with siRNA in LA‐N‐1 cells. *N* = 4. Bottom panel: PTHLH and MYCN protein expression analysis in LA‐N‐1 si*PTHLH* cells 24 and 48 h after transient transfection, respectively. Blots shown are representative of *N* = 3. (C) Relative mRNA expression levels of *PTHLH*,*MYCN*,*CCND1,* and different *MYCN‐*related genes in LA‐N‐1 sh‐*PTHLH* xenografts. Genes were normalized relative to one shNT‐derived xenograft (shNT5) used as a reference. Error bars represent SEM. *P <* *0.05, **P <* *0.01, ***P <* *0.001, ****P <* *0.0001, Mann–Whitney *U*‐test.

Taking into account that PTHLH has been described to exert part of its actions through MYC, MYCN expression was analyzed upon silencing of PTHLH. Stable shRNA‐mediated silencing of *PTHLH* was associated with approximately 50% reduction of *MYCN* mRNA and protein (Fig. 2A) in LA‐N‐1 cell line, whereas those levels were not altered in IMR5 sh‐*PTHLH* clones (data not shown).

Since a profound *PTHLH* downregulation was not achieved, presumably because it compromises neuroblastoma cell viability, and to further validate *MYCN* downregulation upon *PTHLH* silencing, LA‐N‐1 cells were transiently transfected with *PTHLH* small interfering RNA (siRNA). As shown in Fig. 2B, both *PTHLH* and *MYCN* expression levels decreased in si*PTHLH* transfected cells (70% and 30%, respectively) compared with siNT control cells after 24 h of transfection. Moreover, at this time point, PTHLH expression was also reduced, while MYCN protein decay 48 h following transfection (around 44% and 50%, respectively).

Furthermore, when the tumors generated in mice from LA‐N‐1 sh‐*PTHLH* derivatives were analyzed, a significant decreased expression of *PTHLH* and *MYCN* mRNA was seen (Fig. 2C), as it occurred in their cells of origin. In addition, other described target of *MYCN* function such as the anti‐apoptotic *BCL2* gene was significantly repressed. The same tendency was observed with *CCND1* and the inhibitor of differentiation 2 *(ID2);* however, it was not statistically significant. Contrarily, expression of pro‐apoptotic *BAX,* the marker of G1 arrest *CDKN1A* (*p21)*, the senescence‐related gene *CDKN2A (p16)* and *TGFB1* mRNA were upregulated in sh‐*PTHLH* xenografts compared with shNT tumors (Fig. 2C).

### 3.4 EGFR stimulates PTHLH production in neuroblastoma cells

Given that CaSR has been shown to stimulate PTHLH production in other cellular contexts, we initially assessed whether this was also the case in neuroblastoma. Acute CaSR activation did not induce *PTHLH* upregulation in neuroblastoma cell lines exhibiting endogenous CaSR expression (Fig. S2a).

**Figure 3:**
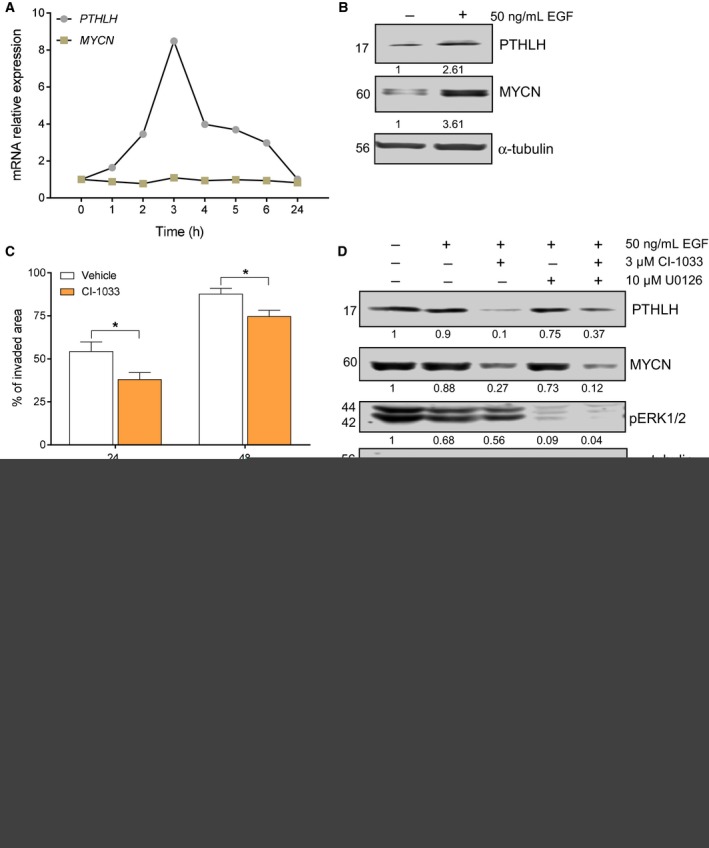
Epidermal growth factor receptor stimulates PTHLH and MYCN expression in neuroblastoma cells. (A) *PTHLH* and *MYCN* mRNA relative expression levels in LA‐N‐1 cells treated with human 10 ng**·** mL ^−1^ EGF or vehicle for the indicated times. (B) PTHLH and MYCN protein expression in LA‐N‐1 cells treated with 50 ng**·** mL ^−1^ EGF or vehicle for 4 h. Band intensities were normalized relative to α‐tubulin. Blots shown are representative of at *N* = 3. (C) Wound healing assay conducted using LA‐N‐1 cells treated with 3 μm CI‐1033 or vehicle. Wound area relative to time 0 was calculated at 24 and 48 h later. *N* = 3. Error bars represent SEM. *P <* *0.05, Mann–Whitney *U*‐test. (D) PTHLH, MYCN, and pERK1/2 protein levels analyzed by immunoblots in LA‐N‐1 cells pretreated with CI‐1033 for 30 min before exposure to EGF and CI‐1033 or U0126 for 3.5 h. Blots shown are representative of *N* = 3. (E) Expression of pEGFR, EGFR, pERK1/2, ERK1/2, pAkt, and Akt in LA‐N‐1 cells pretreated with CI‐1033 for 30 min before exposure to EGF, CI‐1033, U0126, or LY294002 for 5 min. Band intensities normalized relative to α‐tubulin. Blots shown are representative of *N* = 2.

We previously hypothesized that epidermal growth factor (EGF), the main regulator of *PTHLH* in several cancer types, might control PTHLH in neuroblastoma as well. Not surprisingly, expression of *EGFR* was detected in all neuroblastoma cell lines examined (Fig. S2b). Next, neuroblastoma cells were stimulated with human EGF. As expected, exposure to EGF increased *PTHLH* mRNA (Figs 3A and S2c) and protein levels (Fig. 3B) in a time‐dependent manner. Interestingly, EGF stimulus was also associated with upregulation of MYCN protein (Fig. 3B) but not at mRNA level (Fig. 3A). Moreover, neuroblastoma cells were exposed to the EGFR irreversible inhibitor canertinib (CI‐1033) after analyzing IC_50_ concentrations (Table S4) and cell viability was inhibited at low micromolar concentrations. In addition, LA‐N‐1 cells exposed to canertinib exhibited a significant reduction in their migratory capacity (Fig. 3C).

To further assess whether the EGFR family is a key regulator of PTHLH, neuroblastoma cells were pretreated with a low dose of canertinib for 30 min before they were stimulated with human EGF. As shown in Fig. 3D, PTHLH protein expression decreased following treatment with the EGFR irreversible inhibitor. In accordance with this result, canertinib reduced *PTHLH* mRNA expression levels in all neuroblastoma cells analyzed as well (Fig. S2c). Interestingly, reduction of PTHLH levels was associated with decreased expression of MYCN (Fig. 3D).

Moreover, EGF‐stimulated production of PTHLH and MYCN was preceded by increased phosphorylation of EGFR, ERK1/2, and Akt (Fig. 3E). Phosphorylation of these signaling molecules was necessary for EGF‐mediated increase in PTHLH and MYCN levels as inhibitors of both MEK (U0126) (Fig. 3D) and PI3K/Akt (LY294002) (data not shown) blocked their expression. Simultaneous exposure to canertinib and MEK inhibitor blocked this pathway more efficiently than either one drug alone, as evidenced by the significant reduction of PTHLH, MYCN, and phospho‐ERK 1/2 protein levels (Fig. 3D).

### 3.5 PTH1R is overexpressed in *MYCN* nonamplified neuroblastic tumors and its knockdown increases neuroblastoma cell migration and invasion

Our previous results indicate that PTHLH plays an important role in the biology and pathogenesis of neuroblastoma. However, as previously reported, PTHLH can act as an autocrine, paracrine, or intracrine factor depending on the cell context. In order to investigate which previous phenotypes are associated with the intracrine PTHLH actions and which mechanisms are triggered through its receptor PTH1R, we explored the expression of PTH1R in this tumoral context.

Firstly, *PTH1R* mRNA expression was analyzed in a cohort of 44 primary neuroblastic tumors obtained at diagnosis at Hospital Sant Joan de Déu, Barcelona. *PTH1R* mRNA was found significantly higher in patients with age at diagnosis < 18 months and in *MYCN* nonamplified neuroblastomas (Table 1), although this was not an independent predictor of outcome in multivariate analyses. In order to avoid this issue, *PTH1R* expression was then analyzed in four databases of neuroblastic tumors available at Gene Expression Omnibus (GEO) Data Sets (Table S1). It was found significantly overexpressed in *MYCN* nonamplified neuroblastic tumors, in patients with age at diagnosis < 18 months, and in those with lower clinical stages (Table 1).

**Table 1:** Association of *PTH1R* mRNA expression with prognostic factors in neuroblastic tumors.

Dataset	Characteristic		Number of patients	*PTH1R* mRNA median	*P* value^a^
HSJD	Age at diagnosis	<18 months	18	864.0	0.038
		≥18 months	26	387.6	
	Clinical stage	1, 2, 3, 4s	29	562.2	0.56
		4	15	531.9	
	Risk group	High risk	18	497.1	0.50
		Low risk	26	590.2	
	Histologic category^b^	Undifferentiated neuroblastoma	8	570.8	0.69
		Differentiating or poorly differentiated neuroblastoma	27	584.1	
		Ganglioneuroblastoma, ganglioneuroma	8	393.1	
	*MYCN* status	Amplified	9	208.7	0.023
		Nonamplified	35	596.3	
GSE16237	Age at diagnosis	<18 months	39	49.2	0.15
		≥18 months	12	40.0	
	Clinical stage	1, 2, 3, 4s	38	48.0	0.49
		4	13	46.5	
	Risk group	High risk	15	33.4	0.12
		Low risk	36	48.8	
	MYCN status	Amplified	7	25.3	0.0075
		Nonamplified	44	49.3	
GSE45547	Age at diagnosis	<18 months	414	294.8	<0.0001
		≥18 months	235	184.9	
	Clinical stage	1, 2, 3, 4s	435	283.3	<0.0001
		4	214	193.4	
	Risk group^c^	High risk	242	186.4	<0.0001
		Low risk	404	291.8	
	MYCN status^c^	Amplified	93	172.8	<0.0001
		Nonamplified	550	269.4	
GSE49710	Age at diagnosis	<18 months	305	8.2	<0.0001
		≥18 months	193	7.4	
	Clinical stage	1, 2, 3, 4s	315	8.2	<0.0001
		4	183	7.2	
	Risk group^c^	High risk	210	7.2	<0.0001
		Low risk	285	8.2	
	MYCN status^c^	Amplified	92	6.7	<0.0001
		Nonamplified	401	8.0	
GSE3960	Age at diagnosis	<18 months	54	10.9	0.0025
		≥18 months	47	7.6	
	Clinical stage	1, 2, 3, 4s	51	9.6	0.059
		4	50	7.9	
	Risk group	High risk	50	7.9	0.059
		Low risk	51	9.6	
	MYCN status	Amplified	20	7.7	0.30
		Nonamplified	81	9.2	

HSJD, Hospital Sant Joan de Déu.

^a^ Mann–Whitney U‐test except in histologic category (Kruskal–Wallis test).

^b^ One patient had unknown histologic category and was excluded of this analysis.

^c^ Some patients on these datasets had unknown risk group/MYCN status and were excluded of the analyses.

Then, PTH1R expression levels were assessed in neuroblastoma cell lines (Fig. S3a). All of them exhibited endogenous PTH1R forms, although it was interesting to note that dimeric, mature forms of the receptor were expressed at low and high levels in IMR5 and LA‐N‐1 cells, respectively. Interestingly, the highly undifferentiated, aggressive SK‐N‐LP and SK‐N‐JD cell lines expressed low levels of expression of PTH1R mature forms.

**Figure 4:**
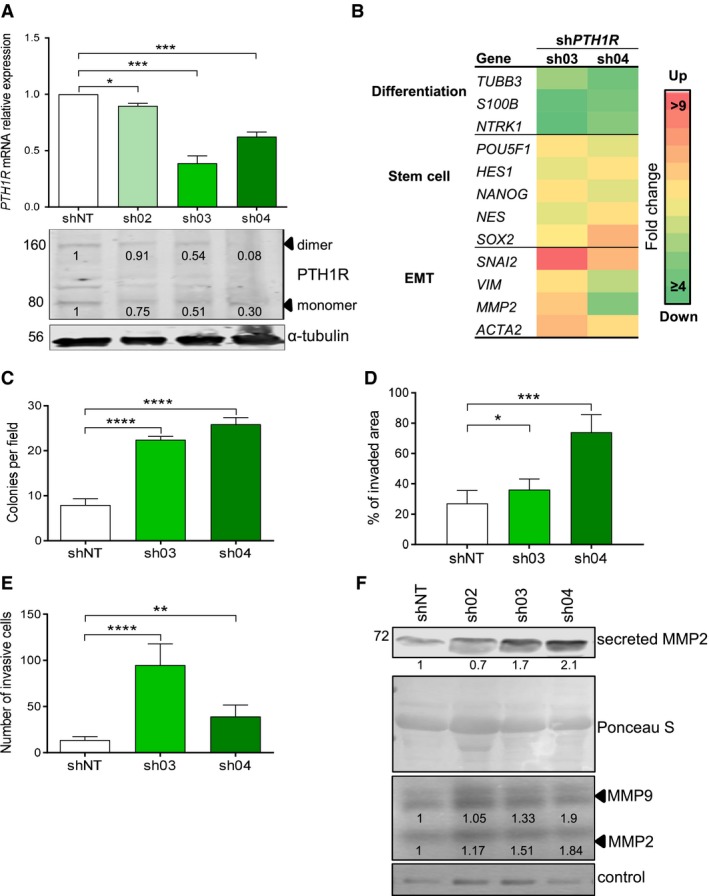
*PTH1R* downregulation increases migration and invasion in neuroblastoma cell lines. (A) *PTH1R* mRNA relative expression and protein levels in LA‐N‐1 cells stably transduced with shRNA against *PTH1R* or a shNT. Band intensities were quantified relative to α‐tubulin and normalized relative to shNT. Data from RT‐qPCR are *N* = 3, and blots shown are representative of *N* = 2. (B) Heatmap of relative mRNA expression levels of specific genes in LA‐N‐1 sh‐*PTH1R*‐derivative cells (sh03, sh04, shNT) determined by RT‐qPCR and normalized relative to shNT. (C) Soft agar colony formation showing anchorage‐independent growth in LA‐N‐1 sh‐*PTH1R*‐derivative cells. Colonies were counted in 10 fields under a 10x objective. Experiments were repeated twice with three replicates for each condition. (D) Wound healing assay conducted in LA‐N‐1 sh‐*PTH1R*‐derivative cells. Wound area relative to time 0 was calculated at 24 h. *N* = 3. (E) Transwell invasion assay with LA‐N‐1 sh‐*PTH1R*‐derivative cells. Invasive cells were counted at 48 h. *N* = 3. (F) Secreted MMP2 protein expression in LA‐N‐1 sh‐*PTH1R*‐derivative cells by western blot. Ponceau S membrane staining was used as the loading control. Blots shown are representative of *N* = 2. Bottom panel: MMP2 and MMP9 activity measured by gelatin zymography analysis. Band intensities were normalized relative to shNT. Representative gels from *N* = 2. Error bars represent SEM. *P <* *0.05, **P <* *0.01, ***P <* *0.001, ****P <* *0.0001, Two‐tailed Student's *t*‐test. EMT: epithelial‐to‐mesenchymal transition.

To functionally address the role of *PTH1R* in neuroblastoma, stable knockdown of the receptor was conducted by shRNA in both LA‐N‐1 and IMR5 cell lines (Figs 4A and S3b, respectively). Stable downregulation of *PTH1R* in these cells resulted in morphological changes, more remarkable in LA‐N‐1 cells. This phenotype change produced large, densely interconnected cell clumps that eventually released as floating spherical cell aggregates (neurospheres) with the capacity to grow in suspension (Fig. S3c,d). Accordingly, these morphological changes were associated with a decrease in neuroblastoma cell differentiation markers and upregulation of epithelial‐to‐mesenchymal transition and stem cell markers (Fig. 4B), thus suggesting a less differentiated stage upon PTH1R downregulation.

To further investigate the functional assessment of PTHLH through its receptor, anchorage‐independent growth, wound healing, and transwell assays were performed in sh‐*PTH1R* derivatives. Downregulation of PTH1R in LA‐N‐1 cells produced a significantly augmented anchorage‐independent growth, migratory, and invasive capacities compared with control cells (shNT) (Fig. 4C–E) without increasing proliferation rates (data not shown). However, IMR5 sh‐*PTH1R* derivatives only exhibited increased invasion capacity relative to shNT cells (Fig. S3e).

Given the relevance of matrix metalloproteinases (MMPs) in migration and invasion, the expression and activity of metalloproteinases 2 and 9 (MMP2 and MMP9, respectively) was evaluated. *MMP2* mRNA and secreted protein levels were significantly higher in LA‐N‐1 sh‐*PTH1R* derivatives compared with the control cells (Fig. 4F). In keeping with these data, increased activity of MMP2 in these cells was also observed (Fig. 4F). In addition, an increased activity of MMP9 in sh‐*PTH1R* derivatives was seen (Fig. 4F) even though *MMP9* mRNA or secreted protein levels were not detectable (data not shown).

Taking advantage of the previously reported phenotypes induced by stable knockdown of *PTH1R* in osteosarcoma, U2OS cell line was simultaneously examined in all these experiments. Consistent with the reported results, U2OS sh‐*PTH1R* derivatives showed decreased migratory and invasive capacities, in sharp contrast with phenotypes observed in neuroblastoma cell lines (Fig. S4).

Taken together, these results indicate that higher expression of *PTH1R* is associated with a more differentiated, less aggressive phenotype, and its downregulation increases neuroblastoma cell migration and invasion *in vitro*.

### 3.6 Intracrine and paracrine actions of PTHLH trigger different phenotypes in neuroblastoma

Surprisingly, stable downregulation of PTHLH and PTH1R in LA‐N‐1 cell line did not produce the same phenotypes, as we could expect from ligand–receptor interaction. While PTHLH was found acting as a growth factor, contributing to the malignant behavior within the tumor growth, invasion, and migration of a *MYCN*‐amplified, *TP53*‐mutated cell line, a higher expression of PTH1R was associated with a less aggressive phenotype.

To further elucidate which actions of PTHLH were PTH1R‐mediated, supplemental assays with addition of the PTH1R‐activating peptide PTHLH (1–34) in the PTHLH‐knockdown scenario were performed.

**Figure 5:**
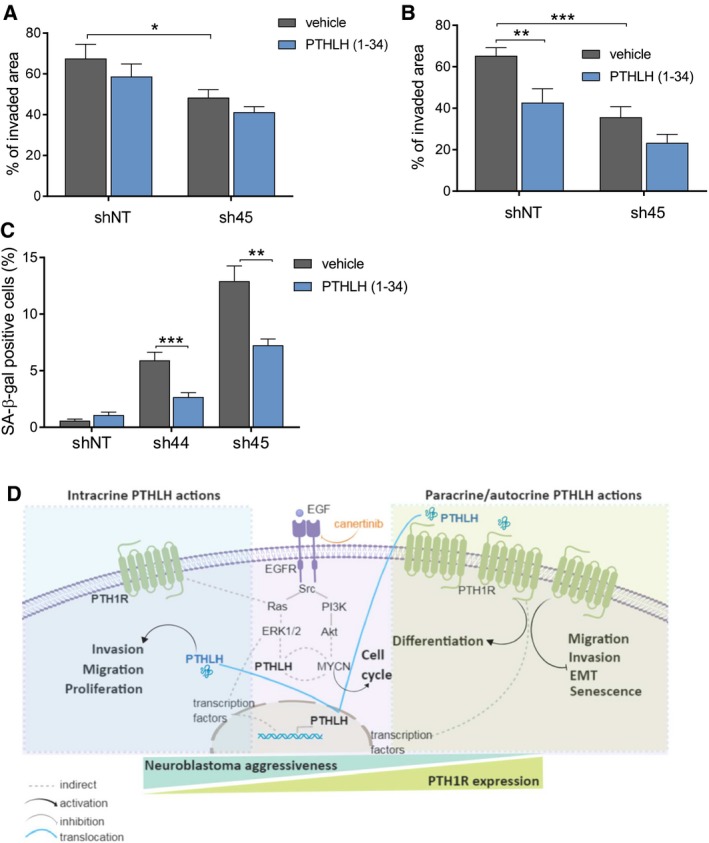
Dual role of PTHLH depending on PTH1R activation. (A) Wound healing assay conducted with LA‐N‐1 sh‐*PTHLH*‐derivative cells (2 × 10^6^) in the presence or absence of 250 nM PTHLH (1–34). Wound area was calculated at 24 h later relative to time 0. *N* = 3. (B) The same wound healing assay performed with IMR5 sh‐*PTHLH*‐derivative cells. *N* = 3. (C) Senescence‐associated β‐galactosidase activity in LA‐N‐1 sh‐*PTHLH*‐derivative cells grown in the presence or absence of 250 nM PTHLH (1–34). Results are expressed as the average percentage of stained cells in ten independent counts. *N* = 3. (D) Model depicting the PTHLH role in neuroblastoma tumorigenicity depending on whether its action is predominantly intracrine (at low levels of PTH1R expression) or paracrine (at high levels of PTH1R expression). Error bars represent SEM. *P <* *0.05, **P <* *0.01, ***P <* *0.001, two‐tailed Student's *t*‐test.

First, no differences in proliferation rate were observed when the PTHLH peptide was added to the media of sh‐*PTHLH* derivatives (data not shown). Then, wound healing assays in LA‐N‐1 and IMR5 sh‐*PTHLH* derivatives with supplementation of the media with the PTHLH (1–34) were performed. As expected, the activation of the PTH1R decreased migration rate both in LA‐N‐1 (Fig. 5A) and IMR5 (Fig. 5B) shNT cell lines. And unexpectedly, the migration in the sh‐*PTHLH* derivatives was even lower when the peptide was added to the media (Fig. 5A,B). This result supports the hypothesis that paracrine and/or autocrine actions of PTHLH through its receptor promote a less aggressive phenotype in neuroblastoma.

On the other hand, whereas LA‐N‐1 sh‐*PHTLH* derivatives showed a significant increase of senescence‐associated β‐galactosidase‐positive cells (Fig. 1F), the addition of PTHLH (1–34) rescued this phenotype (Fig. 5C). This suggests that senescence would be associated to the PTHLH/PTH1R axis functionality.

All these data together reinforce the idea of an oncogenic; intracrine role of PTHLH is acting in more aggressive neuroblastomas, whereas a PTH1R‐dependent, paracrine role of PTHLH would be predominant in good prognosis primary tumors (Fig. 5D).

## 4 Discussion

Malignant neuroblastomas continue to represent a therapeutic challenge. A better understanding of the molecular mechanisms responsible for the phenotypes associated with poor outcome will help to uncover novel, effective treatments. We here investigate the role of PTHLH and PTH1R in neuroblastoma and show their contribution to neuroblastoma behavior, both benign and malignant. Moreover, we have identified one of the factors that controls PTHLH production and provide evidence that its pharmacological regulation might be of therapeutic interest in this group of developmental malignancies.

Our previous findings indicated that PTHLH is expressed in all neuroblastic tumors (de Torres *et al*., 2009). We have now conducted a functional assessment of its role by stable knockdown. This approach has shown that PTHLH acts as a growth factor, necessary for tumor growth, invasion, and migration of a *MYCN*‐amplified, *TP53*‐mutated cell line. Moreover, knockdown of PTHLH decreased MYCN expression, in line with its mechanism of action in other cellular contexts (Sicari *et al*., 2012). These phenotypes were not replicated in IMR5 cells, which are devoid of *TP53* mutation. Interestingly, these cells also show very low levels of PTH1R expression, thus probably facilitating the intracrine activity of PTHLH. Conversely, dimeric, mature forms of PTH1R were highly expressed in LA‐N‐1 cells.

Intracrine actions of PTHLH in cancer cells have been associated with increased resistance to apoptosis and anchorage‐independent growth in human renal cell carcinoma via PI3K/Akt (Agouni *et al*., 2007). Also and in line with our data, *PTHLH* inactivation in human melanoma cells impaired cell motility, invasion, anchorage‐independent growth and reduced their metastatic behavior (Huang *et al*., 2014). PTHLH also induces proliferation of mesangial cells (Hochane *et al*., 2013) and vascular smooth muscle cells (Sicari *et al*., 2012). In both cases, c‐myc upregulation and p27 (Kip1) downregulation were necessary for proliferation to occur. Moreover, and in agreement with our results, PTHLH acts a prosurvival factor in pancreatic cancer cells that up‐regulates c‐myc and increases the ratio of anti‐apoptotic to pro‐apoptotic members of the Bcl2 family (Bhatia *et al*., 2009).

MYCN is a transcription factor that plays a key role in a wide variety of phenotypes associated with malignant behavior of neuroblastomas, including metastatic dissemination (Huang and Weiss, 2013). MYC‐mediated tumorigenesis has been described as a paradigm of oncogene addiction since survival and growth of these tumors depend on the constant overexpression of the oncogene. Accordingly, reduction of MYC expression activates mechanisms that inhibit tumor growth, including senescence (Nardella *et al*., 2011), as we have seen in our models. Although the presence of competent p53 and CDKN2A is considered a requirement for senescence to occur, p53‐independent pathways also exist (Phalke *et al*., 2012; Prieur *et al*., 2011). A senescence‐associated tumor regression following *MYC* inactivation has been reported in several cell contexts (Rakhra *et al*., 2010; Wu *et al*., 2007). We here show that PTHLH and its receptor PTH1R play a role in neuroblastoma cell migration and invasion. Moreover, PTHLH probably exerts these functions, at least in part through regulation of *MYCN*.

Taking into account the therapeutic potential of reducing PTHLH expression in malignant neuroblastomas, we sought to identify the molecule/s that control its production specifically in this tumoral context, as this would provide a therapeutic opportunity. As expected based on our previous reports (de Torres *et al*., 2009), PTHLH was not under the control of the CaSR in neuroblastoma. CaSR acts as a tumor suppressor in this tumoral context, and it is downregulated in malignant neuroblastic tumors. Thus, it was not expected to control the production of a survival factor in these aggressive tumors. Conversely, we here show that PTHLH is controlled by EGFR in neuroblastoma. PTHLH was initially described to be upregulated by epidermal growth factor (EGF) in epithelial cells (Henderson *et al*., 1991). Later, specific inhibition of EGFR expression (Gilmore *et al*., 2009; Nickerson *et al*., 2012) and activity (Cho *et al*., 2004) was reported to notably reduce PTHLH expression in cancer cell lines. Moreover, in neuroblastoma, expression of EGF and EGFR has been associated with aggressive behavior both *in vitro* (Ho *et al*., 2005; Michaelis *et al*., 2008) and *in vivo* (Hossain *et al*., 2012; Richards *et al*., 2010). Interestingly, Hossain and co‐workers showed increased *MYCN* transcription upon EGF stimulation via ERK (Hossain *et al*., 2012). Accordingly, in our models EGF stimulation increased PTHLH expression, as previously reported (Heath *et al*., 1995), and PTHLH induction is associated with higher levels of MYCN. Moreover, irreversible inhibition of EGFR with canertinib reduces ERK1/2 phosphorylation and PTHLH production and induces MYCN downregulation.

Besides its intracrine actions, PTHLH acts as a paracrine factor through PTH1R in several cell contexts (Juppner *et al*., 1988, 1991). For instance, PTHLH exerts opposing functions in vascular smooth muscle cells depending, whereas it acts through its receptor PTH1R or not (Massfelder *et al*., 1997). In neuroblastoma, as our migration assays showed, PTHLH can both promote and inhibit migration depending on whether it acts through its receptor or not. On the other hand, the effects of PTHLH on proliferation (intracrine) and senescence (paracrine) are more easily traceable. In osteosarcoma, in sharp contrast with what we observed in neuroblastoma, PTH1R overexpression has been described to confer increased proliferative, migratory, and invasive capacities (Yang *et al*., 2007). Accordingly, knockdown of PTH1R decreases osteosarcoma invasion and growth, and increases tumor differentiation (Ho *et al*., 2015). Moreover, while normal osteoblasts do not depend on PTHLH for survival, *TP53*‐deficient osteoblasts and osteosarcoma cells undergo apoptosis in the absence of PTHLH (Walia *et al*., 2016). Contrarily, our data indicate that PTH1R expression is higher in the least malignant, *MYCN* nonamplified neuroblastic tumors, and its knockdown promoted increased cell invasion and migration in neuroblastoma cell lines. One of the genetic hallmarks of osteosarcoma is the concomitant loss of function of both *TP53* and *RB1* pathways (Berman *et al*., 2008). The requirement for PTHLH signaling following p53 loss in osteosarcoma initiation and maintenance has been reported elsewhere (Walia *et al*., 2016). However, these pathways are not altered in most neuroblastoma cases at diagnosis, even in high‐risk tumors. These differences might account, at least partially, for the opposite phenotypes promoted by PTH1R in neuroblastoma and osteosarcoma models.

Finally, our results indicating that PTH1R is expressed in the less aggressive neuroblastic tumors, together with our previously reported statistical association of highest levels of PTHLH and Schwannian stroma‐enriched tumors, would be in keeping with evidence provided by Macica *et al*. (2006). These authors showed that both PTHLH and PTH1R are expressed in Schwann cells of dorsal root ganglia and in the sciatic nerve. Moreover, upon sciatic nerve injury, PTHLH is significantly upregulated and promotes a notable increase in the number of Schwann cells.

## 5 Conclusions

Taken together, our data show that downregulation of PTHLH reduces MYCN expression, tumor growth, invasion, and migration in a *MYCN*‐amplified and *TP53*‐mutated neuroblastoma cell lines exhibiting high levels of PTH1R. These phenotypes are not seen in a model with reduced expression of PTH1R. Also, PTH1R knockdown is associated with a more aggressive phenotype, and increases cell migration, invasion, and anchorage‐independent growth in neuroblastoma. On the other hand, PTHLH is highly expressed in well‐differentiated, Schwannian stroma‐rich neuroblastic tumors and high levels of PTH1R expression are associated with MYCN nonamplified, benign neuroblastomas. Altogether, our data would be consistent with the hypothesis that in aggressive, undifferentiated neuroblastic tumors, which express low levels of PTH1R, a preferential intracrine action of PTHLH would be promoted. Whereas in *MYCN* nonamplified, benign neuroblastic tumors, expressing high levels of PTH1R, the paracrine activities of PTHLH would be predominant.

Therefore, given that we have unveiled the factor mainly responsible for PTHLH production in this tumoral context, it might be of therapeutic interest since it is feasible to reduce PTHLH production specifically in malignant neuroblastomas without damaging normal tissues.

## Conflict of interest

The authors declare no conflict of interest.

## Author contributions

MG, CJRH, and SML participated in the design of the study, carried out the experiments, performed statistical analysis, and drafted the manuscript. EGA assisted in the experiments, participated in its design, and contributed to draft the manuscript. SPJ performed statistical analysis and contributed to draft the manuscript. CL and JM provided clinical databases of neuroblastoma patients and contributed to the manuscript. CdT conceived of the study, participated in its design and coordination, and wrote the manuscript. All authors read and approved the final manuscript.

## Supporting information


**Fig. S1.** *PTHLH* downregulation in neuroblastoma cell lines.
**Fig. S2**. EGFR, but not CaSR, stimulates *PTHLH* production in neuroblastoma cells.
**Fig. S3**. *PTH1R* downregulation in neuroblastoma cell lines.
**Fig. S4**. *PTH1R* downregulation in osteosarcoma cells.
**Table S1**. Neuroblastoma databases.
**Table S2.** Primers sequences, Assays‐on‐Demand used for RT‐qPCR and shRNA references.
**Table S3.** Primary antibodies used for immunoblots.
**Table S4.** IC_50_ values of canertinib in neuroblastoma cell lines.
