# Supplementary material for: Parathyroid hormone‐like hormone plays a dual role in neuroblastoma depending on PTH1R expression
Source: Mol Oncol. 2019 Jul 19;13(9):1959–75. doi: 10.1002/1878-0261.12542 (PMC6717746; doi:10.1002/1878-0261.12542)
Supplement: Supplementary file 1 — Fig. S1. PTHLH downregulation in neuroblastoma cell lines. Fig. S2. EGFR, but not CaSR, stimulates PTHLH production in neuroblastoma cells. Fig. S3. PTH1R downregulation in neuroblastoma cell lines. Fig. S4. PTH1R downregulation in osteosarcoma cells. Table S1. Neuroblastoma databases. Table S2. Primers sequences, Assays‐on‐Demand used for RT‐qPCR and shRNA references. Table S3. Primary antibodies used for immunoblots. Table S4. IC50 values of canertinib in neuroblastoma cell lines. [file MOL2-13-1959-s001.pdf]

Supplementary Figure S1

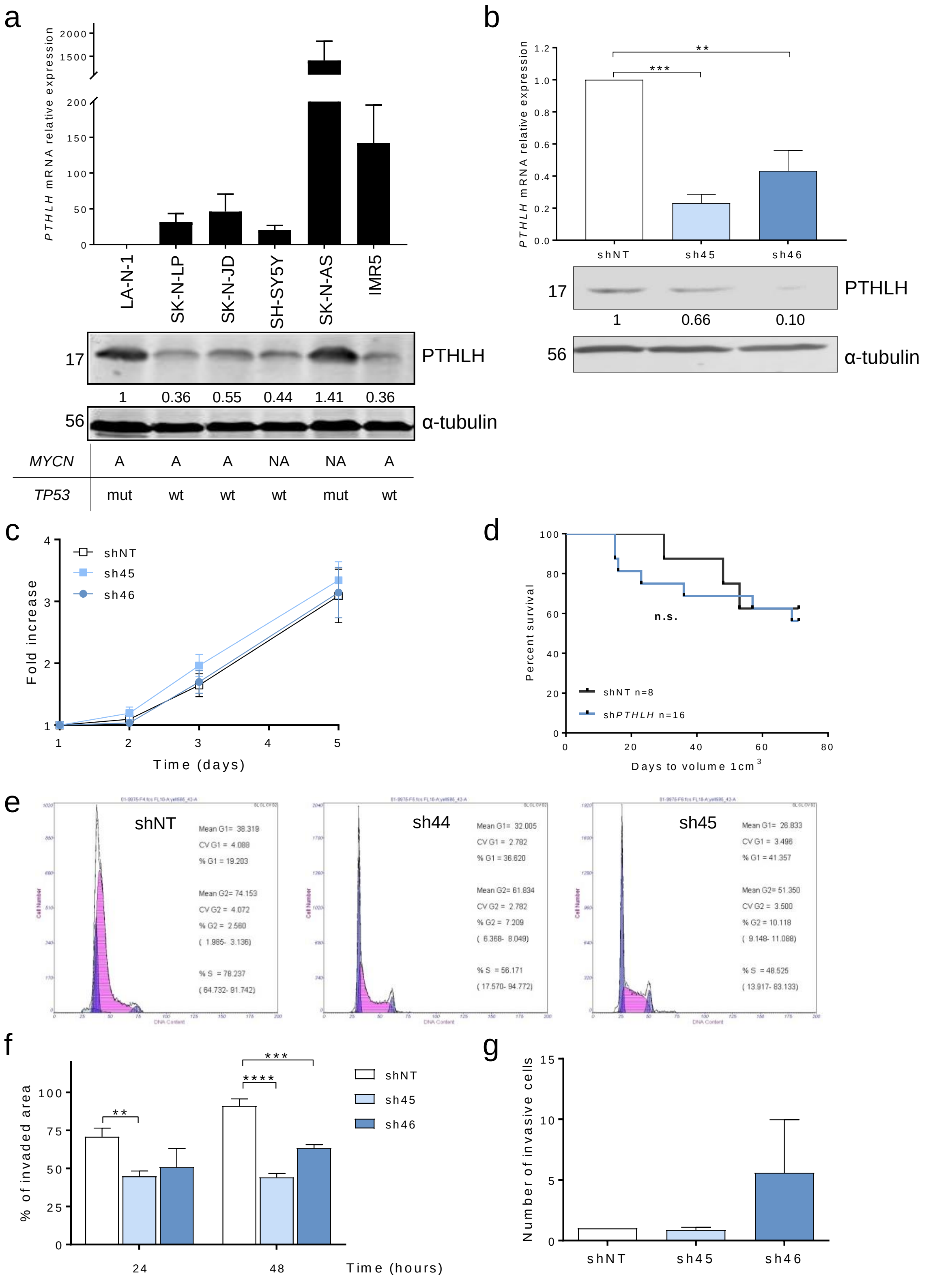

Supplementary Figure S1. *PTHLH* downregulation in neuroblastoma cell lines.

a. *PTHLH* mRNA relative expression and protein levels in neuroblastoma cell lines. Band intensities were quantified relative to α-tubulin. Data from RT-qPCR are N=4 and blots shown are representative of N=3. **b**. *PTHLH* mRNA relative expression and protein levels in IMR5 sh-*PTHLH* derivative cells. Band intensities were quantified relative to α-tubulin and shNT. **c.** Cell viability of IMR5 sh-*PTHLH* derivative cells. N=6. **d.** IMR5 sh-derivative (sh45, sh46 and shNT) cells (10^7) subcutaneously inoculated in four to six-week-old female athymic nude mice. The log-rank statistic was used to compare the tumor time to reach 1 cm^3 between groups. **e.** Cell cycle profiles from a representative experiment with LA-N-1 sh-*PTHLH* derivatives at 8 hours. **f.** Wound healing assay conducted with IMR5 sh-*PTHLH* derivative cells. Wound area relative to time 0 was calculated at 24 and 48 hours later. N=6. **g.** Transwell invasion assay with IMR5 sh-*PTHLH* derivative cells. Invasive cells were counted at 48 hours. N=3. Error bars represents SEM. *P < 0.05, **P < 0.01, ***P < 0.001, ****P < 0.0001, Two-tailed Student's *t*-test. A: amplified; NA: non-amplified; mut: mutated; wt: wild-type.

Supplementary Figure S2

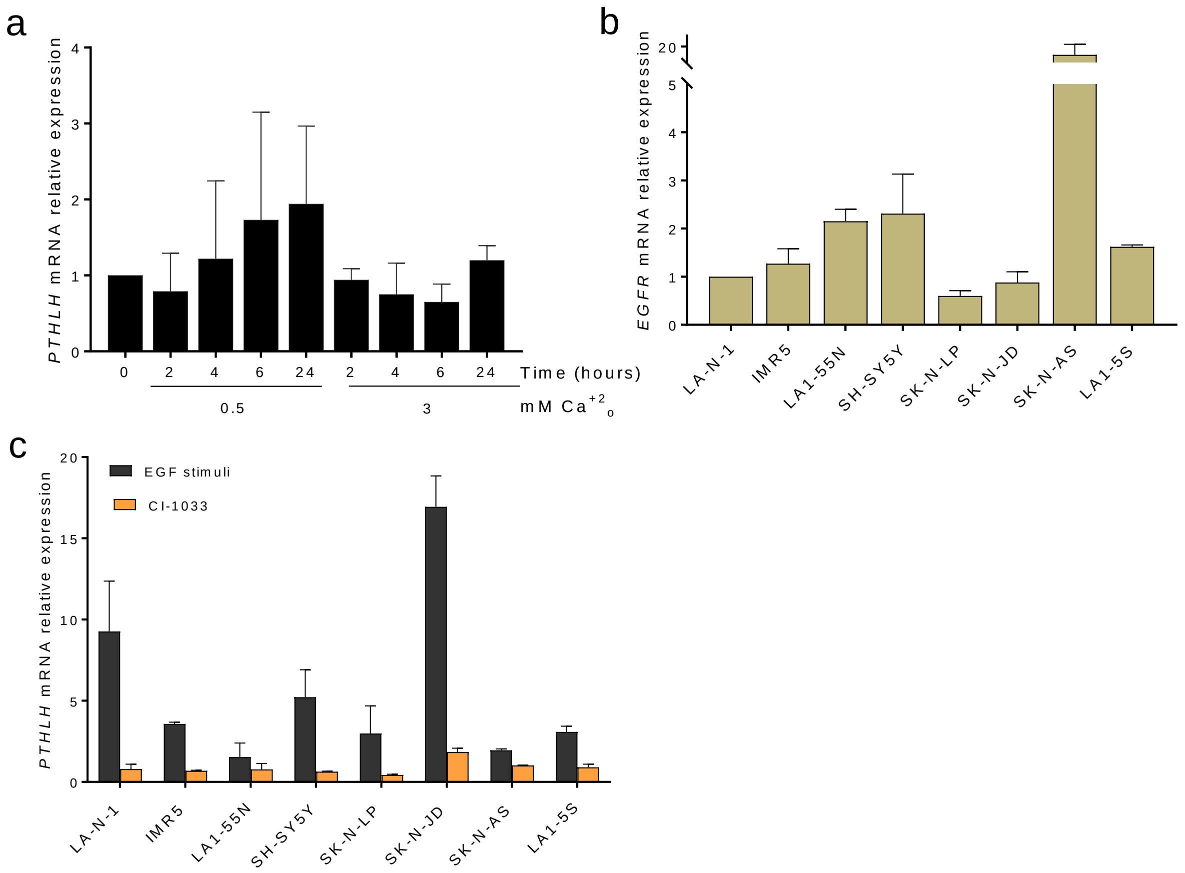

Supplementary Figure S2. EGFR, but not CaSR, stimulates *PTHLH* production in neuroblastoma cells.

a. *PTHLH* mRNA relative expression levels in LA-N-1 cells after acute activation of CaSR, as described in Material and Methods (2.11 section), and normalized relative to time 0. N=3. **b.** *EGFR* mRNA relative expression levels in neuroblastoma cell lines and normalized relative to those detected in LA-N-1 cells. N=5. **c.** *PTHLH* mRNA relative expression levels in neuroblastoma cells treated with 10 ng/mL EGF stimuli or pretreated with CI-1033 for 30 minutes before exposure to EGF or vehicle for 3 hours. Relative expression levels were normalized relative to vehicle and time 0. N=3. Error bars represents SEM. Mann-Whitney *U* test.

Supplementary Figure S3

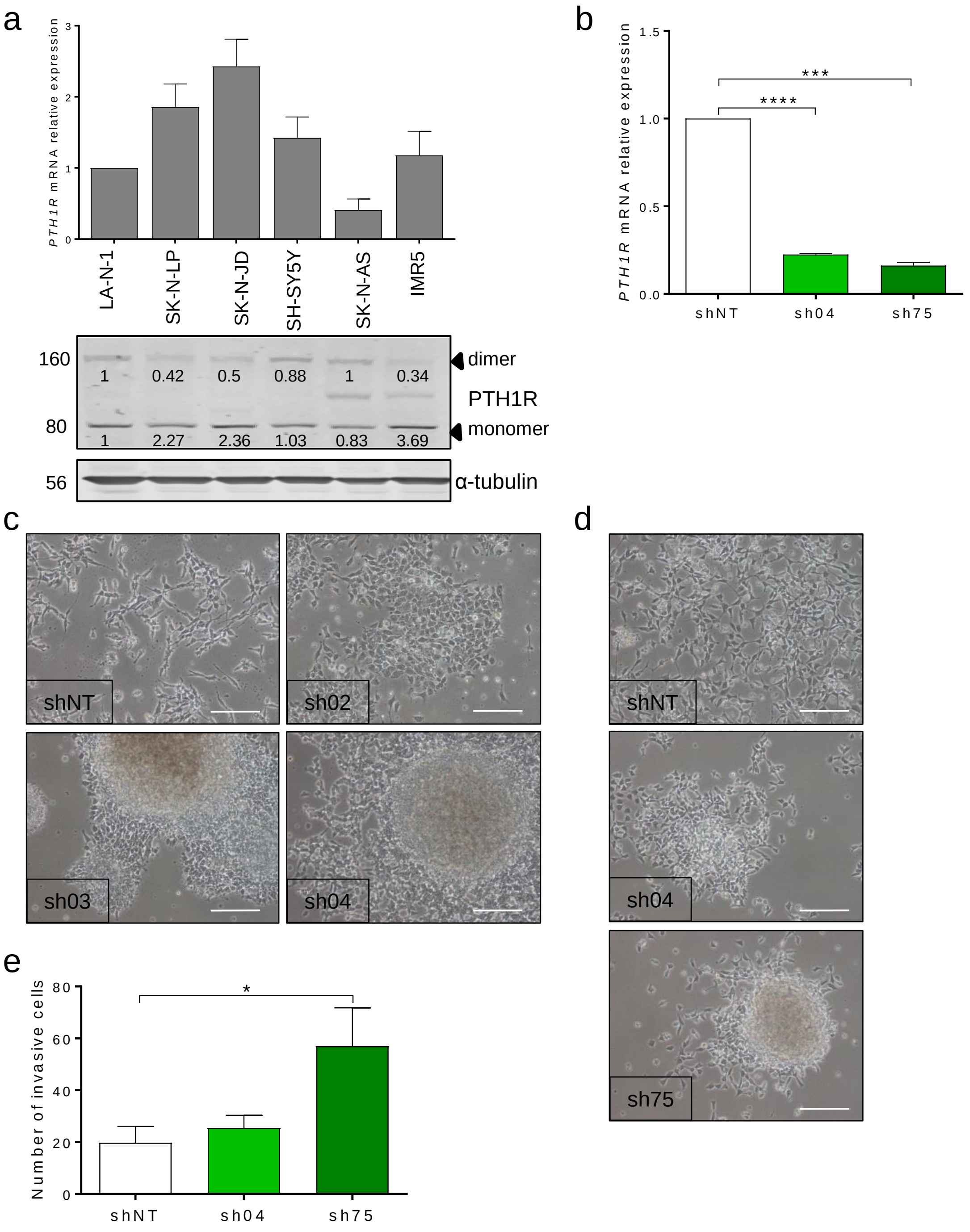

Supplementary Figure S3. ***PTH1R* downregulation in neuroblastoma cell lines**.

a. *PTH1R* mRNA relative expression and protein levels in neuroblastoma cell lines. Dimer and monomer isoforms were quantified relative to α-tubulin and normalized relative to LA-N-1 expression. Data from RT-qPCR are N=4 and blots shown are representative of N=2. **b.** *PTH1R* mRNA relative expression levels in IMR5 sh-*PTH1R* derivative cells **c.** Phase-contrast images of LA-N-1 cells showing morphological changes induced upon *PTH1R* downregulation (magnification, x10; scale bars: 135 µm). **d.** Phase-contrast images of IMR5 cells upon *PTH1R* downregulation (magnification, x10; scale bars: 135 µm). **e.** Transwell invasion assay performed with IMR5 sh-*PTH1R* derivative cells. N=3. Error bars represents SEM. *$P < 0.05$, ***$P < 0.001$, ****$P < 0.0001$, Two-tailed Student's *t*-test.

Supplementary Figure S4

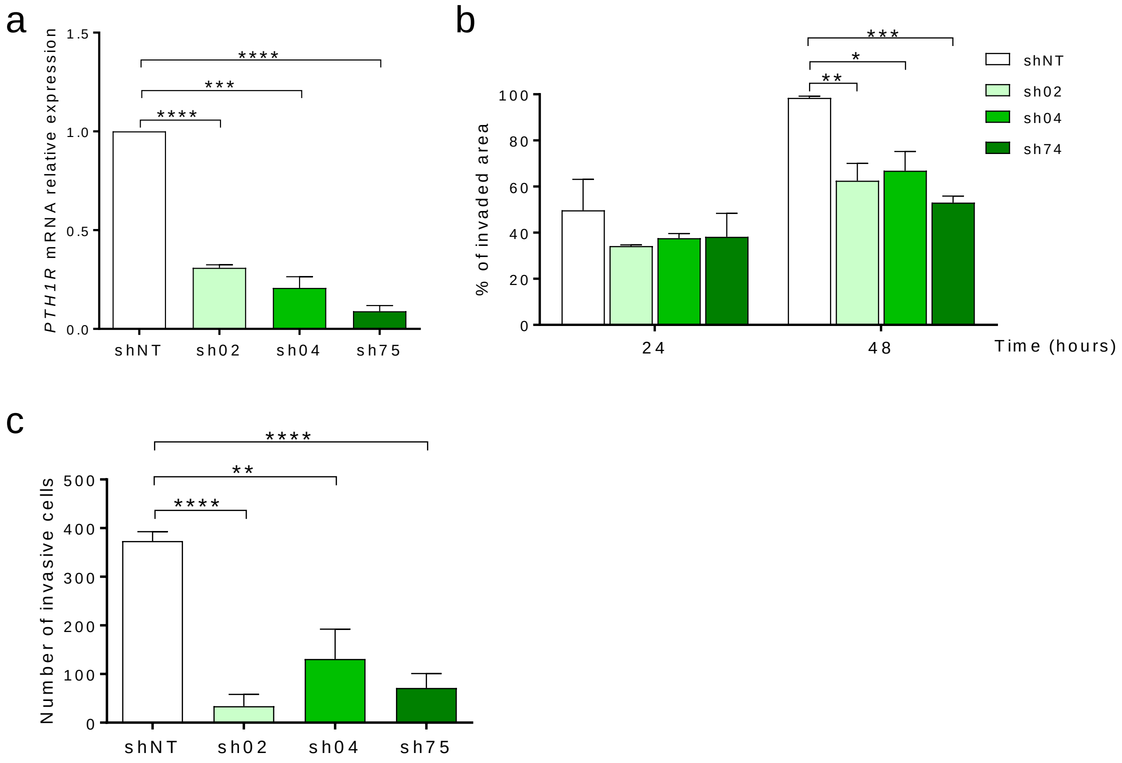

Supplementary Figure S4. ***PTH1R* downregulation in osteosarcoma cells**.

a. *PTH1R* mRNA relative expression levels in U2OS osteosarcoma cells stably transduced with sh-*PTH1R* or a shNT. Results are expressed as the average of N=3 and normalized relative to shNT. **b.** Wound healing assay conducted with U2OS sh-*PTH1R* derivative cells. Migration rates were quantified by measuring four different wound areas at 24 and 48 hours (magnification, x10). N=2. **c.** Transwell invasion assay with U2OS sh-*PTH1R* derivatives cells. N=2. Error bars represents SEM. *$P < 0.05$, **$P < 0.01$, ***$P < 0.001$, ****$P < 0.0001$, Two-tailed Student's *t*-test.

Supplementary Table S1. Neuroblastoma Databases

GEO ID	Title	Cohort	Link	References
**GSE16237**	*Expression data of human neuroblastoma tissue samples*	51	https://www.ncbi.nlm.nih.gov/geo/query/acc.cgi?acc=GSE16237	Ohtaki *et al.* 2010
**GSE45547**	*Hox-C9 activates the intrinsic pathway of apoptosis and is associated with spontaneous regression in neuroblastoma*	649	https://www.ncbi.nlm.nih.gov/geo/query/acc.cgi?acc=GSE45547	Kocak *et al.* 2013
**GSE49710**	*RNA-Seq reveals an unprecedented complexity of the neuroblastoma transcriptome and is suitable for clinical endpoint prediction*	498	https://www.ncbi.nlm.nih.gov/geo/query/acc.cgi?acc=GSE49710	C. Wang *et al.* 2014<br>SEQC/MAQC-III Consortium. 2014<br>S. Munro *et al.* 2014<br>Z. Su *et al.* 2014
**GSE3960**	*Classification of neuroblastoma by integrating gene expression pattern with regional alterations in DNA copy number*	101	https://www.ncbi.nlm.nih.gov/geo/query/acc.cgi?acc=GSE3960	Q. Wang *et al.* 2006<br>Balamuth *et al.* 2010<br>K. Wang *et al.* 2011

Supplementary Table S2. Primers sequences, Assays-on-Demand used for RT-qPCR and shRNA references

Gene Symbol	Primer forward (5'-3')	Primer reverse (5'-3')	Assay on Demand
ACTA2	ccgcgatctcaccgactacc	gggccacgtagcacagcttc	
BAX	tgacatgttttctgacggcaac	ggaggcttgaggagtctcacc	
BCL2	tgtggagagcgtcaaccgggag	caagctcccaccagggccaaact	
CASR			Hs01047793_m1
CCND1	ccgagaagctgtgcatctaca	aggttccacttgagcttgttcac	
CDKN1A	ggacagcagaggaagaccatgt	tggagtggtagaaatctgtcatgc	
CDKN2A	atggagccttcggctgactg	gcgctgcccatcatcatgac	
HES1	gaagcacctccggaacct	gtcacctcgttcatgcactc	
ID2	tcctgtccttgcaggcttctg	tcagccacacagtgctttgc	
MMP2	caaggagtacaacagctgcactgata	ggtgcagctctctcatatttgttgc	
MYCN			Hs00232074_m1
NANOG	tcccgagaaaagattagtcagca	agtggggcacctgtttaactt	
NES	ctgctacccttgagacacctg	gggctctgatctctgcatctac	
NTRK1			Hs01021011_m1
POU5F1	gaaacccacactgcagatca	cggttacagaaccacactcg	
PTH1R			Hs00174895_m1
PTHLH			Hs00174969_m1
S100B			Hs00902901_m1
SNAI2	aacgcctccaaaaagccaaa	ggttgtggtatgacaggcatgg	Hs00950344_m1
SOX2	tgctgcctctttaagactaggac	cctggggctcaaacttctct	
TBP	gaacatcatggatcagaacaacag	attggtgttctgaataggctgtg	
TGFβ1	cccacaacgaaatctatgac	tgtatttctggtacagctcc	
TUBB3			Hs00801390_m1
VIM	tacaggaagctgctggaagg	accagagggagtgaatccag	

Gene	shRNA Reference	Abbreviation used
PTHLH	TRCN0000083843	sh43
	TRCN0000083844	sh44
	TRCN0000083845	sh45
	TRCN0000083846	sh46
PTH1R	TRCN0000003302	sh02
	TRCN0000003303	sh03
	TRCN0000003304	sh04
	TRCN0000003305	sh05
	TRCN0000010775	sh75
**Non-Target**	SHC002	shNT

Supplementary Table S3. Primary antibodies used for immunoblots

Primary Antibody (clone)	Company	Cat No	Type[a]	Dilution
Akt (pan) (C67E7)	Cell Signaling	#4691	MoAb	1:1000
Calcium Sensing Receptor	Fisher Scientific	PA1-37213	PoAb	1:500
EGF Receptor (D38B1)	Cell Signaling	#4267	MoAb	1:1000
MMP-2 (D8N9Y)	Cell Signaling	#13132	MoAb	1:1000
MYCN (NCM II 100)	Millipore	OP13	MoAb	1:500
p44/42 MAPK (ERK1/2) (137F5)	Cell Signaling	#4695	MoAb	1:1000
Phospho-Akt (S473) (D9E)	Cell Signaling	#4060	MoAb	1:1000
Phospho-EGF Receptor (Y1068) (D7A5) XP	Cell Signaling	#3777	MoAb	1:1000
Phospho-p44/42 MAPK (ERK1/2) (T202/Y204) (D13.14.4E)	Cell Signaling	#4370	MoAb	1:1000
PTH/PTHLH Receptor Antibody (3D1.1)	Millipore	#05-517	MoAb	1:750
PTHLP (Ab-2) (34-53)	Millipore	PC09	PoAb	1:250

[a]MoAb: monoclonal antibody; PoAb: polyclonal antibody

Supplementary Table S4. IC_{50} values of canertinib in neuroblastoma cell lines

Cell line	IC_{50} Canertinib (μM)
**LA-N-1**	3.9
**LA1-55N**	7.8
**IMR5**	3.5
**SH-SY5Y**	3.8
**SK-N-JD**	3.8
**SK-N-LP**	3.9
**LA1-5S**	6.8
**SK-N-AS**	6.3
